# Supplementary Feed Additives Can Improve Lamb Performance in Terms of Birth Weight, Body Size, and Survival Rate

**DOI:** 10.3390/ani13060993

**Published:** 2023-03-08

**Authors:** Leila Ahmadzadeh-Gavahan, Ali Hosseinkhani, Valiollah Palangi, Maximilian Lackner

**Affiliations:** 1 Department of Animal Science, Faculty of Agriculture, University of Tabriz, Tabriz 5166616471, Iran; 2 Department of Animal Science, Faculty of Agriculture, Ege University, Bornova, Izmir 35100, Türkiye; 3 Department of Industrial Engineering, University of Applied Sciences Technikum Wien, Hoechstaedtplatz 6, 1200 Vienna, Austria

**Keywords:** feed restriction, monensin sodium, offspring performance, placental characteristics, propylene glycol, rumen-protected choline chloride

## Abstract

**Simple Summary:**

Grazing in arid and semi-arid regions causes ewes to suffer from feed shortages to some degree. This is particularly problematic in late pregnancy, when maximum fetal growth has been reached and the nutritional requirements of the growing fetus exceed maternal nutrient intake. The result is weaker offspring with lower birth weights and survival rates, which in turn affects livestock profitability. Finding an alternative to improve the supply of necessary nutrients to dams during pregnancy will help farm managers achieve healthy lambs with higher survival rates. Using additives involved in the energy status of animals, this study has shown that administration of these additives during late gestation reverses the debilitating effects of feed restriction and improves placental efficiency, thereby enhancing nutrient delivery to the growing fetus, such that lambs from ewes offered diets containing these additives had higher birth weights and subsequently higher survival rates. These results will help livestock producers in arid and semi-arid regions to benefit from the combined administration of the three substances, namely propylene glycol, rumen-protected choline chloride, and monensin sodium, when feeding pregnant ewes.

**Abstract:**

To evaluate the effects of supplementation of feed additives in the last trimester of pregnancy on placental characteristics and offspring performance, this study was conducted with 48 estrous-synchronized Ghezel ewes that had randomly been assigned to one of the following six groups (*n* = 8): ad libitum feeding (AL); feed restriction (RF; 60% of ad libitum intake); feed restriction + propylene glycol (PG); feed restriction + propylene glycol + monensin sodium (MS); feed restriction + propylene glycol + rumen-protected choline chloride (RPC); feed restriction + propylene glycol + monensin sodium + rumen-protected choline chloride (PMC). Birth weight, body size, and rectal temperature of lambs were determined within 24 h of birth. The presence of lambs at 87 days of age was used as an index of survival to weaning. The outcome of this study was that the average placental weight of ewes in the AL and MS groups was the highest and lowest, respectively, among the treatment groups (*p* < 0.01). RPC ewes presented higher placental efficiency compared to AL, RF, and MS ewes (*p* < 0.05). The largest and smallest crown-to-rump lengths (CRLs) were observed in PMC and RF lambs, respectively (*p* < 0.01). In addition, lambs born from PMC, RPC, and PG ewes had a longer curved crown-to-rump length (CCRL) than those born from AL and RF ewes (*p* < 0.01). The concurrent administration of propylene glycol and rumen-protected choline chloride resulted in the highest birth weight among treatment groups (*p* < 0.01). Lambs born to PMC and RPC ewes had a higher survival rate and rectal temperature than those born to RF ewes (*p* < 0.05). It can be concluded that although dietary restriction does not have adverse effects on lambs’ performance compared with ad libitum intake, the combined administration of propylene glycol and rumen-protected choline chloride in the ewes’ restricted diet can improve placental characteristics and subsequently amend lambs’ birth weight and body size. Therefore, the combined administration of these additives can be practiced during feed restriction.

## 1. Introduction

Survival of lambs to weaning is a critical factor in the profitability of sheep production systems and is influenced by several factors. One of the most important factors affecting lamb survival to weaning is ewe nutrition during pregnancy [1,2]. In semi-arid regions such as Iran, pasture grazing provides most of the nutritional needs of pregnant ewes. In the early months of pregnancy, this is not problematic because the animals are at the maintenance level. In the later months of pregnancy, however, pregnant ewes must accept some degree of feed restriction caused by higher nutrient requirements of the ewe and the growing fetus, as this period usually coincides with lower quality and quantity of pastures. These disruptions in maternal feed intake can have different consequences for the ewes and their offspring. For example, maternal undernutrition in late gestation is known to reduce lamb survival rate directly through lower colostrum production, resulting in weaker maternal ewe–lamb bonding [3], and also indirectly through lower lamb birth weight [4,5,6]. The effect of maternal dietary restriction on lamb birth weight may also be mediated by the regulation of fetoplacental unit development [5,7]. During mid to late pregnancy, the placenta grows faster and undergoes some structural and morphological transformations, increasing its functional capacity to provide nutrients to the developing fetus [8]. Because of the positive correlation between placental size and fetal weight in late pregnancy [9], a decrease in placental size results in a decrease in offspring birth weight [10]. Lower birth weight, in turn, leads to a reduction in body size and organogenesis, as a strong correlation between birth weight and pathology has been previously demonstrated [11].

In late pregnancy, feeding supplements that increase the nutrient supply to the dam, and thus to the growing fetus, may mitigate the negative effects of maternal dietary restriction. Feed additives such as propylene glycol, monensin sodium, and choline chloride can improve blood glucose levels [12] and the energy status of ewes in several ways, including increasing dietary energy concentration, altering rumen fermentation to produce more propionate [13], and improving hepatic lipid metabolism. Since the fetus has a limited ability for glucose synthesis as the main substrate for fetal metabolism, the use of these supplements may promote fetal growth and development. This may promote viability. 

Propylene glycol (1,2-propanediol, CAS No. 57-55-6) is an approved feed additive according to Commission Regulation (EU) No. 68/2013 of 16 January 2013, on the Catalog of Feed Materials (No. 13.11.1).

Monensin (CAS No. 17090-79-8, CAS No. 22373-78-0 for sodium salt) was previously approved in the EU as a feed additive to improve growth and feed efficiency in cattle, excluding lactating cows (Council Regulation (EC) No. 1831/2003). This use in cattle was phased out in January 2006. Although monensin sodium is safe and effective in target species when used at the recommended dosages [14], poisoning can occur following incorrect feed mixing, misidentification of the recipient, excessive dosage in the diets of target species, and use in non-recommended species [15,16,17,18]. The toxicity of monensin to cattle and other species is well documented. The lethal dose 50 (LD_50_) of monensin is 2–3 mg/kg body weight for horses [19], 21.9 mg/kg for cattle [20], and 11.9 mg/kg for sheep [21]. Monensin is an antibiotic that is isolated from *Streptomyces cinnamonensis.*

Choline chloride (CAS No. 67-48-1) is a vitamin substitute often included in feed additives for poultry, fish, and swine. There are insufficient data to support precise estimates of maximum tolerated dietary levels of choline chloride. However, published information suggests that tolerance for choline is high in most species. Choline chloride is listed in the Code of Federal Regulations as a nutrient and/or dietary supplement that is generally recognized as safe [22]. There are no data on the safety of choline chloride in small ruminants; however, in a study with dairy cows, choline chloride was found to be safe even at relatively high levels of supplementation (326 g/head/day) and did not cause animal health problems [23]. 

The effects of restricted maternal diet and supplementation with feed additives that affect energy metabolism, such as propylene glycol, monensin sodium, and rumen-protected choline chloride, on placental development, birth weight, and offspring survival are not known. We hypothesized that reductions in offspring birth weight, body size, or survival resulting from dietary restriction would be related to reductions in placental size or efficiency. Therefore, the present study aimed to determine how dietary restrictions and the administration of energy metabolism-related feed additives during late pregnancy affects placental growth and development and offspring performance.

## 2. Materials and Methods

This study was approved by the Biomedical Ethics Committee of the University of Tabriz (Approval code: 2048) and was performed in compliance with the guideline for the care and use of laboratory animals in Iran [24]. It builds upon the work of Ahmadzadeh-Gavahan and Hosseinkhani (2022) on feed restriction and supplementation with propylene glycol, monensin sodium, and rumen-protected choline chloride in periparturient Ghezel ewes [13].

### 2.1. Animals, Treatments, and Management

This study was conducted at the University of Tabriz’s agricultural research farm. Ninety-six 2–3-year-old Ghezel ewes were chosen to be evaluated in the present study. Ewes were estrous synchronized using progesterone sponges (ESPONJAVET, Hipra, Spain) inserted into the vagina for 14 days. Afterward, the sponges were removed, and 48 h later all ewes were naturally mated with rams. Only the ewes that showed no signs of a second estrus 17–20 days after mating and became pregnant after the first estrus were further evaluated. Therefore, forty-eight 2–3-year-old Ghezel ewes (body weight (BW) = 65.53 ± 6.90 kg; body condition score (BCS) = 3.17 ± 0.56) were involved in this experiment (these ewes and this experiment are those whose data and results have been published in part in previous articles [12,13]). After mating, the ewes were allowed to graze on their native rangeland. Pregnancy was confirmed by ultrasonography (CTS-900 V Neo, Shantou Institute of Ultrasonic Instruments (SIUI) Co., Shantou, China) on the 40th day of pregnancy, and an additional ultrasound was conducted on day 90 of pregnancy to determine the litter size. During a two-week adaptation period, the animals’ dry matter intake requirement was estimated based on the difference between the feed offered to the ewes the previous day and the feed refusal, which resulted in approximately 10% of the total feed offered being refused [25]. By day 105 of pregnancy, the animals were moved to large straw-bedded pens in the barn and randomly assigned to six groups as follows (*n* = 8): ad libitum feed intake (AL, control); restricted feeding (RF, 60% of ad libitum intake); restricted feeding plus propylene glycol (67 g/ewe/day; PG); restricted feeding plus propylene glycol and monensin sodium (30 mg/ewe/day; MS); restricted feeding plus propylene glycol and rumen-protected choline chloride (6 g/ewe/day; RPC); restricted feeding plus propylene glycol, monensin sodium, and rumen-protected choline chloride (PMC). Experimental additives including monensin sodium (Monensin-Behrood; containing 10% monensin sodium), propylene glycol (PROPYPACT 55; containing 45% propylene glycol), and rumen-protected choline chloride (VETACHOL; containing 25% coated choline chloride) were provided by Behrood Atrak Co. Arak, Iran; Difagri Co., Montaigu-Vendée, France; and SILA Co., Noale, Italy, respectively. The levels of propylene glycol, monensin sodium, and rumen-protected choline chloride used in this study were based on the manufacturer’s guidelines and previously published studies in dairy ewes and goats [26,27,28]. Water and salt licks were freely available to all animals. The feed allocated to ewes was balanced based on litter size, BW, and gestation stage [11] according to the recommendations of the National Research Council [29] at a forage-to-concentrate (F: C) ratio of 65:35 (Table 1) [13]. The ad libitum fed ewes received their meals at 08:30, 11:00, and 16:00 daily, while the restricted ewes were offered feed at 08:30 and 16:00. After lambing, these diets were withdrawn from the experimental groups and all ewes were offered ad libitum feeding during lactation.

Feed and refusal samples were collected daily at 08:30 h, homogenized, subsampled, and stored at −20 °C for chemical analysis. After oven drying at 55 °C for 72 h and grinding through a Wiley mill 1 mm sieve (Arthur H. Thomas Co., Philadelphia, PA, USA), composite samples were analyzed for dry matter, crude protein, calcium, and phosphorus content according to AOAC procedures [30].

### 2.2. Placental Evaluation

Immediately after parturition, all placentas were collected, and their weight was determined after washing. Then the placentomes were pooled and counted, and the morphological type of each placentome was recorded. The morphological type was determined using the classification scheme of Vatnick et al. [31] and the appearance of the placentome, as follows: (A) the cotyledonary tissue is completely surrounded by caruncular tissue; (B) the cotyledonary tissue grows over the surrounding caruncular tissue; (C) the cotyledonary tissue is on one side and caruncular tissue is on the other, resulting in flat placentomes; (D) placentomes are completely everted and resemble bovine placentomes. Placental efficiency was calculated as the grams of lambs produced per gram of placenta from each ewe [32]. The number of cotyledons per gram of placental weight was used to calculate the cotyledon density [33].

### 2.3. Lamb Measurements

Crown-to-rump length (CRL; the distance from the crown of the skull to the base of the tail), curved crown-to-rump length (CCRL; the distance from the crown of the skull to the end of the rump, measured along the backbone), abdomen girth, heart girth (the distance around the rib cage directly behind the forelegs), head girth, left foreleg and left hindleg girth, rump height, withers height, pin bone width, and hook bone width were measured with a cloth tape to determine body size within 24 h of birth.

The birth weight of lambs was also determined immediately after birth. The presence of lambs at postnatal day 87 was used as an index of survival until weaning, and the survival rate was calculated as the proportion of live lambs present at weaning to all lambs identified at birth.

The rectal temperature of newborn lambs was measured with a clinical thermometer before suckling. This was repeated 24 h and 72 h after birth.

### 2.4. Statistical Analysis

Statistical analysis was performed in a completely randomized design using the Statistical Analysis System (SAS v.9.3; SAS Institute Inc., Cary, NC, USA). The UNIVARIATE procedure and the Shapiro–Wilk test were used to first check the normality of the data. If the probability of the Shapiro–Wilk test was equal to or greater than 0.05, the data were assumed to be normally distributed. 

The lamb-related parameters, including birth weight and body size, were analyzed using the residual maximum likelihood approach with a GLM procedure, whereas repeated measurements of lamb rectal temperature at each time point were analyzed using the MIXED procedure of SAS. The GENMOD procedure with logit as a link function (binomial distribution and logit link function) was used to analyze the survival rate. The survival rate is a binomial trait and is called an “all or none” trait. This trait is coded as 1 (success: if the lamb was born alive and survived to weaning age) and 0 (failure: if the lamb was born alive but died before reaching weaning age). The output of the GENMOD procedure is the logit value and can be expressed as a mean ± SEM or back-transformed percentage. During back-transforming, the predicted probability equals $\frac{e^{n}}{1+e^{n}}$. The model included ewe dietary treatment and parity, lamb sex, and litter size as fixed effects and lamb body weight as a covariate. For analysis of lamb rectal temperature, fixed effects of recording time and the interaction between maternal dietary treatments and recording time were included in the model. 

All the placenta-related parameters, including placental weight, total placentome number, cotyledon density, and percentage of each morphologic type of placentome, were analyzed using the GLM procedure. The percentage of morphological type of placentomes was calculated as the number of each placentomal type (i.e., A, B, C, or D) divided by the total number of placentomes for that conceptus. The model included the fixed effects of ewe dietary treatment, parity, and litter size. Further, the model included the effects of ewe BW and BCS as covariates. 

All systematic effects that were not significant (*p* > 0.05) were removed from the model. All data are expressed as least-square means ± standard errors. When differences were found, the PDIFF option of the Tukey–Kramer test was used to compare the least-square means.

## 3. Results

### 3.1. Placental Characteristics

Ewes in the AL and MS groups revealed the highest and lowest mean placental weight among the experimental groups, respectively (*p* < 0.01), whereas the other groups were intermediate (Table 2). Placental efficiency was improved in the RPC ewes when compared with AL, RF, and MS ewes (*p* < 0.05), but their efficiency was similar to that of the PMC and PG ewes (*p* > 0.05). In addition, RF ewes had the lowest placental efficiency among all treatment groups (*p* < 0.05). Similar to placental weight, ewes in the AL group numerically had the highest total placentome number (*p* < 0.01), while ewes in the RF and MS groups had the lowest total placentome number (*p* < 0.01). The results of the data analysis also showed that cotyledon density was not affected by the different dietary treatments (*p* > 0.05).

**Figure 1:**
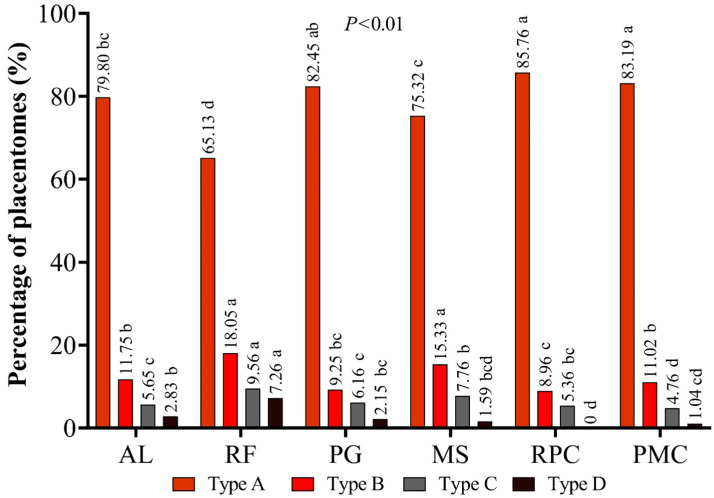
Effect of feed restriction and supplementation with the 3 compounds propylene glycol, monensin sodium, and rumen-protected choline chloride in late pregnancy on the percentage of type A, B, C, and D placentomes. AL = ad libitum feed intake; RF = restricted feeding; PG = restricted feeding plus propylene glycol; MS = restricted feeding plus propylene glycol and monensin sodium; RPC = restricted feeding plus propylene glycol and rumen-protected choline chloride; PMC = restricted feeding plus propylene glycol, monensin sodium, and rumen-protected choline chloride. Values are means with their standard errors being shown by vertical bars. Different letters indicate significant differences among treatments.

The effects of the experimental treatments on placentomal morphology are shown in Figure 1. Ewes in the PMC and RPC groups had predominantly type A placentomes compared with those in the AL, RF, and MS groups (*p* < 0.01), whereas the percentage of this type of placentome was similar in ewes from the PG, PMC, and RPC groups (*p* > 0.05). The highest percentage of type B placentomes was found in the placentas of RF and MS ewes (*p* < 0.01), with RF and PMC ewes having the highest and lowest percentage of type C placentomes, respectively (*p* < 0.01). In addition, feed restriction without supplementation led to the highest percentage of type D placentomes among all treatment groups (*p* < 0.01).

### 3.2. Lamb Measurements

Table 3 summarizes the effects of late gestational maternal dietary treatments on lamb measurements. Lambs born to RPC ewes had the greatest circumference of the left foreleg and hindleg among the treatment groups (*p* < 0.01), although the circumference of the left hindleg was not significantly different from that of PMC lambs (*p* > 0.05). Lambs from PMC, RPC, and PG ewes had greater rump height at birth than those from RF ewes (*P* < 0.01), whereas those from AL and MS ewes had intermediate height. Lambs born from RPC, PMC, and PG groups had longer withers height than the other three groups (*p* < 0.01). Regarding abdominal girth, PMC lambs had the largest mean value among the treatment groups (*p* < 0.01), although the difference with RPC lambs was not significant (*p* < 0.01). The lowest abdominal girth was found in lambs born to RF ewes (*p* < 0.01), followed by AL, MS, and PG lambs.

Combined administration of propylene glycol, monensin sodium, and rumen-protected choline chloride resulted in a larger heart girth compared to AL, RF, and PG treatments (*p* < 0.01), with lambs from RF ewes having the smallest heart girth (*p* < 0.01). The results also showed that lambs from PMC and RPC ewes had a larger head circumference than those from RF, PG, and MS ewes (*p* < 0.01). 

Lambs born from PMC ewes had a higher CRL than lambs born from AL, RF, and MS ewes (*p* < 0.01), while this parameter was similar in lambs born from PMC, RPC, and PG ewes (*p* > 0.05). In this way, lambs born from RF ewes had the shortest CRL value (*p* < 0.01). On the other hand, lambs born from PMC, RPC, and PG ewes had longer mean CCRL measurements than those born from AL and RF ewes (*p* < 0.01), and the shortest CRL was found in RF lambs (*p* < 0.01). In addition, lambs born from RPC and PMC ewes had wider pin and hook bones than those born from RF and MS ewes (*p* < 0.01), while their differences from PG and AL lambs were not significant (*p* > 0.05).

### 3.3. Offspring Performance

**Figure 2:**
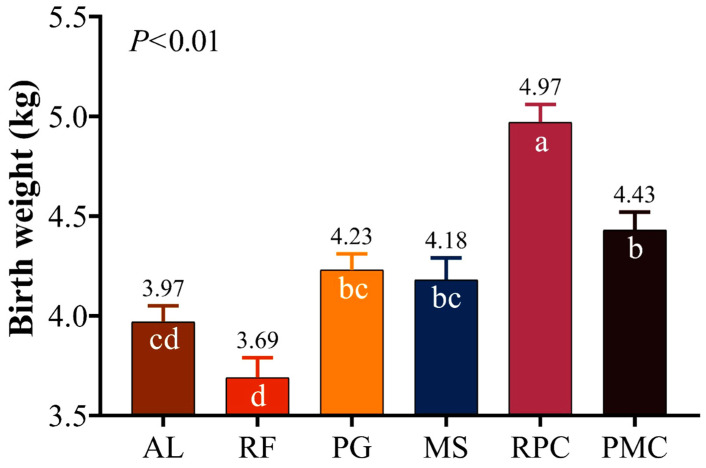

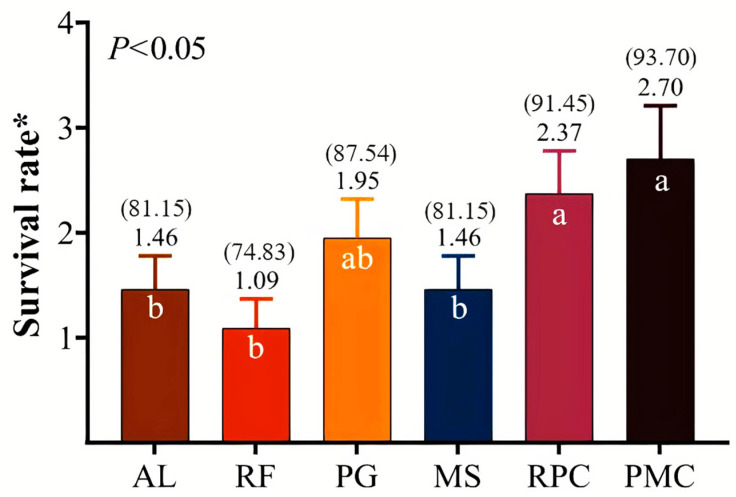
Effect of feed restriction and supplementation with propylene glycol, monensin sodium, and rumen-protected choline chloride in late pregnancy on birth weight (kg) and survival rate (%) of lambs. AL = ad libitum feed intake; RF = restricted feeding; PG = restricted feeding plus propylene glycol; MS = restricted feeding plus propylene glycol and monensin sodium; RPC = restricted feeding plus propylene glycol and rumen-protected choline chloride; PMC = restricted feeding plus propylene glycol, monensin sodium, and rumen-protected choline chloride. Values are means with their standard errors being shown by vertical bars. Means with different letters indicate significant differences among treatments. * Data are shown as logit values in columns and back-transformed percentages in parentheses.

The combined supplementation of propylene glycol and rumen-protected choline chloride to the restricted diet of ewes resulted in the highest birth weight among the treatment groups (Figure 2) (*p* < 0.01). Lambs born from PMC ewes were heavier at birth than those born from AL and RF ewes (*p* < 0.01). At the same time, the body weight of lambs born from PG and MS ewes was better than that of lambs born from RF ewes (*p* < 0.01). The numerically lowest birth weight was found in lambs born from RF ewes, and it was not significantly different from that of AL lambs (*p* > 0.05).

The results of the analysis showed that lambs born from PMC and RPC ewes had a higher survival rate until weaning than those born from MS, RF, and AL ewes (Figure 2) (*p* < 0.05), while the survival rate of PG lambs was comparable to these two categories.

### 3.4. Rectal Temperature

**Figure 3:**
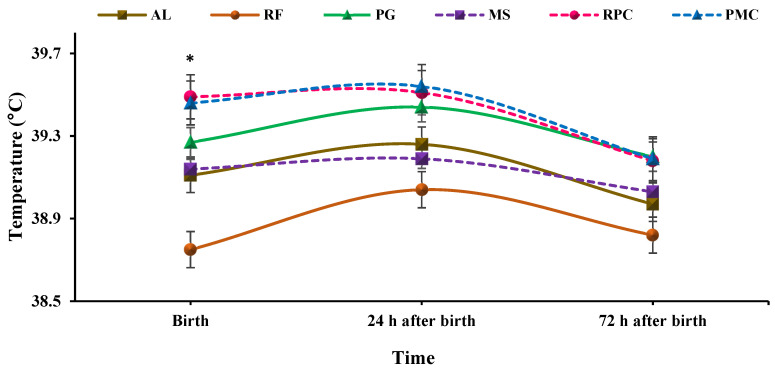
Effect of feed restriction and supplementation with propylene glycol, monensin sodium, and rumen-protected choline chloride in late pregnancy on the rectal temperature of lambs during the first three postnatal days. AL = ad libitum feed intake; RF = restricted feeding; PG = restricted feeding plus propylene glycol; MS = restricted feeding plus propylene glycol and monensin sodium; RPC = restricted feeding plus propylene glycol and rumen-protected choline chloride; PMC = restricted feeding plus propylene glycol, monensin sodium, and rumen-protected choline chloride. Values are means with their standard errors being shown by vertical bars. Mean values differ significantly at each time point: * *p* < 0.05.

The results of the effects of maternal nutritional treatments on the rectal temperature of the offspring are given in Figure 3. On the first day after birth, the lambs of RPC and PMC ewes had a significantly higher rectal temperature than the lambs of RF ewes (*p* < 0.05), whereas the rectal temperature of the lambs of the other groups was intermediate. During the second and third days of life, maternal dietary treatments had no significant effect on the rectal temperature of the offspring (*p* > 0.05).

## 4. Discussion

### 4.1. Placental Characteristics

In this study, feed restriction resulted in lower placental weight compared with ad libitum feed intake, which is in line with the results of Kelly [34] and Clarke et al. [35] who applied the dietary restriction between days 40 and 100 or between days 30 and 80 of pregnancy. However, Lekatz et al. [10] reported that nutrient restriction during mid and late pregnancy did not significantly affect cotyledon, caruncle, and placental weights. Contrary to the findings of Cristofolini et al. [36] about an increase in the placentome number during dietary restriction (70% dietary restriction) of Anglo-Nubian goats, we detected a compromised total placentome number via feed restriction. This lower placentome number in restrictively fed ewes was associated with reduced placental efficiency in these ewes, as the direct correlation between the number of placentomes and placental efficiency has been previously established. 

Considering the negative correlation between placental weight and placental efficiency (r = –0.67) as previously pointed out by Cristofolini et al. [36], we also detected higher placental efficiency in response to feed restriction. The higher placental efficiency in RF ewes caused the weight of lambs born from these ewes to be similar to that of lambs born from AL ewes (Figure 2). This may be due to the fact that larger placentas are less effective at transferring nutrients to the fetus because they need and consume more nutrients than smaller ones, which ultimately leads to lower birth weight [11]. Therefore, the lower placental weight in RPC ewes was associated with their higher efficiency, as the highest lamb birth weights were found in this group (Figure 2). Additionally, this higher placental efficiency, as well as birth weight, in RPC ewes may be related to the rumen-protected choline chloride since it has already been shown that choline chloride can alter inflammatory reactions, oxidative stress, and apoptosis of placental trophoblast cells [37]. The role of choline chloride can be mediated by betaine, which reduces osmotic pressure [38] and improves placental efficiency. Moreover, the higher placental efficiency of PG, MS, RPC, and PMC ewes compared with RF ewes may be due to the higher nutrient supply, especially glucose, since an increase in serum glucose level by administration of these three compounds has been previously reported [12].

Although the main growth of the placenta occurs during mid-gestation [39], individual placentomes may undergo some functional and morphological transformation as the nutritional needs of the fetus increase [40]. Results from various laboratories demonstrated that in restrictively fed ewes compared to their adequately fed counterparts, type A placentomes are converted to type B, C, or D placentomes [41]. This indicates the ability of the ovine placenta to adapt to reductions in nutrient intake, which will be associated with the flattening and heaviness of placentomes [42] and thereby improved nutrient delivery to the fetus [43]. In other words, as sheep placentomes shift from less advanced type to more advanced type (from type A to type D placentomes), their size and angiogenesis [44] as well as their blood flow increase [42], leading to more nutrient delivery and rescuing growth-restricted fetuses. In this way, we found that feed restriction led to an increase in the percentage of type C and D placentomes, which comes in the same line as the findings of Kelly [34]. In this study, the combined administration of propylene glycol, monensin sodium, and rumen-protected choline chloride reduced the percentage of advanced placentomes, which may be related to the increased availability of nutrients when taking these supplements [13]. Therefore, placentomal conversion did not occur due to sufficient nutrient availability.

### 4.2. Lamb Measurements

Prepartum maternal feed restriction affected the body measurements of the offspring. This is in agreement with the results of Pillai et al. [45] who found that maternal under- or overfeeding at days 45, 90, and 135 of gestation altered body size and organ growth in relation to the stage of gestation, such that the average heart circumference at the birth of lambs of the restrictively fed ewes was smaller than that of the other groups. Laporte-Broux et al. [25] also demonstrated that kids from restrictively fed goats had a smaller abdominal girth due to maternal feed restriction in the third trimester of gestation. Such a disturbance in the morphological characteristics of the offspring as a result of feed restriction could be related to the lower birth weight of lambs from restrictively fed ewes, since a positive correlation between birth weight and pathology has been previously established [11]. Moreover, it is clear that there is a positive relationship between placental efficiency and lamb morphological characteristics (r^2^ = 50.3) [11], and an increase in nutrient flow through the placenta leads to an increase in body size. Considering the increased placental efficiency (Table 2) and the previously noted increase in glucose and insulin levels in response to the administration of propylene glycol, monensin sodium, and rumen-protected choline chloride [12], which affect the growth and development of the offspring, administration of these additives to the ewes was expected to improve the conformation indices of the lambs.

### 4.3. Offspring Performance

Since the fetal body grows by approximately 80% during the last six weeks of gestation, any maternal nutritional restriction during this period leads to intrauterine growth restriction, which retards the growth of the fetal body [46,47]. In the present study, dietary restriction during the last six weeks of gestation did not significantly reduce the birth weight of the offspring compared with ad libitum feeding. Martin et al. [48] also reported that maternal food restriction in mid-to-late pregnancy has no significant effect on fetal weight, which is consistent with our findings. However, some researchers have reported a decrease in offspring birth weight through 60% dietary restriction [10,45,49] or restriction of energy and protein intake [50,51] during late pregnancy. These contradictory results could be related to the timing and duration of dietary restriction and the presence of maternal fat reserves to compensate for dietary restriction [52]. However, mobilization of maternal reserves during dietary restriction has a negligible role in compensating for nutrient deficiencies, since the placenta may transfer fewer long-chain fatty acids, non-esterified fatty acids, and ketone bodies, limiting the direct use of maternal energy reserves for fetal growth and development and leading to a decrease in birth weight [53].

Glucose is the main energy source for embryonic growth; therefore, the high rate of entry of amino acids and glucose into cells leads to increased metabolism and growth of the fetus. Since the administration of propylene glycol, monensin sodium, and rumen-protected choline chloride leads to an increase in glucose concentration, as previously established [12], an increase in the birth weight of the offspring at the time of taking these supplements was expected, which is consistent with the findings of Smith et al. [54] and Li et al. [55]. Since lambs born to RPC ewes had higher birth weights than those born to PG ewes, it can be concluded that the higher birth weight may be partly due to choline chloride consumption. In this way, King et al. [56] demonstrated that choline supplementation during pregnancy in mice increased fetal weight, which was associated with an increase in placental weight, body length, and placental efficiency. 

In this study, the lowest survival rate was numerically observed in lambs born to RF ewes. This could be related to the lower birth weight of these lambs. Lightweight lambs are born with limited energy reserves and lose more heat to the environment than heavier lambs due to their larger surface area and are more susceptible to hypothermia. Furthermore, lightweight lambs are weaker at birth and take more time to stand and ingest colostrum [57]. Since the survival rate of lambs depends on their ability to stand and consume colostrum quickly at birth (due to the nutritional and immunological benefits of colostrum), maternal undernutrition plays a very important role in altering the vitality of the offspring and their survival rate through reducing birth weight or the availability and production of milk or colostrum for the offspring [58]. 

### 4.4. Rectal Temperature

Since the development of fetal brown adipose tissue is influenced by the maternal diet in late pregnancy [59], any alteration to the maternal diet at this time may affect the temperature balance of the offspring’s body. In this study, although lambs from restrictively fed ewes had similar rectal temperatures to lambs from ewes fed ad libitum, dietary restriction numerically reduced the rectal temperature of the offspring. This result was in line with the reports of Ekpe and Christopherson [60] and Moore et al. [61], who found a decrease in offspring rectal temperature as a result of maternal dietary restriction. This may be partly related to the immaturity of brown adipose tissue and a decrease in thermogenesis function [56]. In addition, the rectal temperature of lambs may be influenced by their birth weight since Clarke et al. [62] showed that lambs with lighter birth weights had limited adipose tissue at birth, making them less able to maintain body temperature in cold environments. Accordingly, the high rectal temperature of lambs from RPC and PMC groups can be explained by their higher birth weight or the higher placental efficiency of the dams. For a discussion on feed restriction and supplementing with propylene glycol, monensin sodium, and rumen-protected choline chloride in periparturient Ghezel ewes, see [13].

## 5. Conclusions

In conclusion, this study revealed that monensin sodium resulted in the lightest placentas, while the combined supplementation of propylene glycol and rumen-protected choline chloride to the restricted diet of ewes improved the reproductive and growth performance of ewes and lambs. Therefore, to achieve optimal performance of Ghezel ewes and their offspring during feed restriction, especially in semi-arid regions such as Iran, the simultaneous administration of propylene glycol and rumen-protected choline chloride without monensin sodium, which may have potential risk factors, is recommended. 

## Figures and Tables

**Table 1:** Formulation and chemical composition of the basal diet ^1^. Same data as reported in [13].

Item	Diet Composition	
	Prepartum	Postpartum
Ingredients (g/kg DM)		
Corn silage	330	200
Alfalfa hay	190	290
Wheat straw	140	110
Ground barley grain	160	132
Wheat bran	122	150
Molasses	50	60
Soybean meal	0	50
Salt	3	3
Mineral–vitamin premix ^2^	5	5
Chemical composition		
Dry matter (% of fresh weight)	73	78.90
Metabolizable energy ^3^ (MJ/kg DM)	9.37	9.66
Crude protein (g/kg of DM)	100.6	127.0
Calcium (g/kg of DM)	4.37	5.48
Phosphorus (g/kg of DM)	3.5	4.1
Dry matter intake (kg/day)	2.4	1.9

^1^ Reproduced with permission from Ahmadzadeh-Gavahan and Hosseinkhani [13]. ^2^ Ingredients per kg included 500,000 IU vitamin A, 100,000 IU vitamin D3, 100 mg vitamin E, 196,000 mg calcium, 96,000 mg phosphorous, 19,000 mg magnesium, 46,000 mg sodium, 2000 mg manganese, 3000 mg iron, 300 mg copper, 3000 mg zinc, 100 mg cobalt, 100 mg iodine, 1 mg selenium, and 400 mg butylated hydroxytoluene oxide. ^3^ Estimated using values obtained from the NRC (2007).

**Table 2:** Effect of feed restriction and supplementation with the 3 compounds propylene glycol, monensin sodium, and rumen-protected choline chloride in late pregnancy on placental characteristics.

Item	AL	RF	PG	MS	RPC	PMC	*p* Value
Placental weight (g)	781.6 ± 23.2 ^a^	587.5 ± 22.2 ^b^	592.7 ± 24.5 ^b^	477.3 ± 25.0 ^c^	638.1 ± 23.8 ^b^	591.7 ± 20.0 ^b^	<0.01
Placental efficiency	10.5 ± 0.5 ^b^	8.2 ± 0.5 ^c^	11.3 ± 0.6 ^ab^	10.7 ± 0.5 ^b^	12.8 ± 0.5 ^a^	10.9 ± 0.4 ^ab^	<0.05
Placentome number	75.9 ± 2.1 ^a^	57.5 ± 1.8 ^c^	66.8 ± 2.0 ^b^	58.5 ± 2.3 ^c^	68.4 ± 2.2 ^ab^	72.6 ± 2.2 ^ab^	<0.01
Cotyledon density	0.1 ± 0.006	0.12 ± 0.005	0.12 ± 0.006	0.13 ± 0.006	0.12 ± 0.006	0.11 ± 0.005	NS

AL = ad libitum feed intake; RF = restricted feeding; PG = restricted feeding plus propylene glycol; MS = restricted feeding plus propylene glycol and monensin sodium; RPC = restricted feeding plus propylene glycol and rumen-protected choline chloride; PMC = restricted feeding plus propylene glycol, monensin sodium, and rumen-protected choline chloride. ^a b c^ Means within rows with different superscripts are significantly different.

**Table 3:** Effect of feed restriction and supplementation with the 3 compounds propylene glycol, monensin sodium, and rumen-protected choline chloride in late pregnancy on lambs’ measurements (cm) at birth.

Item	AL	RF	PG	MS	RPC	PMC
Left hind-leg girth	8.7 ± 0.1 ^b^	8.6 ± 0.1 ^b^	8.7 ± 0.1 ^b^	8.8 ± 0.1 ^b^	9.4 ± 0.1 ^a^	9.0 ± 0.1 ^ab^
Left fore-leg girth	8.5 ± 0.1 ^b^	7.9 ± 0.1 ^b^	8.4 ± 0.1 ^b^	8.4 ± 0.1 ^b^	8.8 ± 0.1 ^a^	8.6 ± 0.1 ^b^
Rump height	39.0 ± 0.2 ^ab^	38.0 ± 0.2 ^b^	40.0 ± 0.19 ^a^	39.0 ± 0.2 ^ab^	39.8 ± 0.2 ^a^	39.5 ± 0.2 ^a^
Withers height	35.4 ± 0.2 ^b^	34.2 ± 0.2 ^b^	36.9 ± 0.2 ^a^	35.5 ± 0.2 ^b^	37.1 ± 0.2 ^a^	37.0 ± 0.2 ^a^
Abdomen girth	41.3 ± 0.2 ^c^	39.0 ± 0.3 ^d^	42.7 ± 0.3 ^bc^	41.4 ± 0.3 ^c^	43.8 ± 0.3 ^ab^	44.4 ± 0.2 ^a^
Heart girth	39.3 ± 0.3 ^b^	36.6 ± 0.3 ^d^	36.8 ± 0.3 ^c^	40.2 ± 0.3 ^ab^	41.2 ± 0.3 ^ab^	41.7 ± 0.3 ^a^
Head girth	20.1 ± 0.1 ^ab^	19.0 ± 0.1 ^c^	19.4 ± 0.1 ^bc^	19.1 ± 0.1 ^c^	20.3 ± 0.1 ^a^	20.6 ± 0.1 ^a^
CRL	50.3 ± 0.3 ^c^	47.5 ± 0.3 ^d^	53.1 ± 0.3 ^ab^	51.6 ± 0.3 ^bc^	52.2 ± 0.3 ^abc^	53.8 ± 0.3 ^a^
CCRL	31.6 ± 0.2 ^b^	28.9 ± 0.2 ^c^	34.0 ± 0.2 ^a^	32.7 ± 0.2 ^ab^	33.3 ± 0.2 ^a^	34.0 ± 0.2 ^a^
Pin bone width	10.8 ± 0.1 ^ab^	9.7 ± 0.1 ^b^	10.2 ± 0.1 ^ab^	9.6 ± 0.1 ^b^	10.4 ± 0.1 ^a^	10.6 ± 0.1 ^a^
Hook bone width	12.2 ± 0.1 ^abc^	11.7 ± 0.1 ^c^	12.4 ± 0.1 ^ab^	11.8 ± 0.1 ^bc^	12.5 ± 0.1 ^a^	12.9 ± 0.1 ^a^
Number of lambs	11	12	12	11	12	13

AL = ad libitum feed intake; RF = restricted feeding; PG = restricted feeding plus propylene glycol; MS = restricted feeding plus propylene glycol and monensin sodium; RPC = restricted feeding plus propylene glycol and rumen-protected choline chloride; PMC = restricted feeding plus propylene glycol, monensin sodium, and rumen-protected choline chloride. ^a b c d^ Means within rows with different superscripts are significantly different: *p* < 0.01.

## Data Availability

The datasets generated during and/or analyzed during the current study are available from the corresponding author (A.H.) on reasonable request.
